# Driving change: exploring cattle transporters’ perspectives to improve worker and animal well-being

**DOI:** 10.1093/tas/txaf021

**Published:** 2025-02-12

**Authors:** Paxton A Sullivan, Angela Varnum, Libby Bigler, M Caitlin Cramer, I Noa Román-Muñiz, Lily N Edwards-Callaway

**Affiliations:** Department of Animal Sciences, Colorado State University, Fort Collins, CO 80521, USA; Private practice veterinarian, Buffalo, MN 55313, USA; Department of Animal Sciences, Colorado State University, Fort Collins, CO 80521, USA; Department of Animal Sciences, Colorado State University, Fort Collins, CO 80521, USA; Department of Animal Sciences, Colorado State University, Fort Collins, CO 80521, USA; Department of Animal Sciences, Colorado State University, Fort Collins, CO 80521, USA

**Keywords:** cattle welfare, focus groups, job satisfaction, social sustainability, transportation, training and education

## Abstract

The cattle industry has made considerable efforts to adopt more sustainable beef production practices. Still, the social pillar of sustainability—especially workforce well-being—often receives less attention than the environmental and economic pillars. There is also limited information about the perspectives of U.S. cattle transporters, who play a critical role in ensuring animal welfare during the final stages of the beef supply chain. This study explored cattle transporters’ perspectives on management practices related to cattle care and well-being, training, work environment, and community. Cattle transporters (N = 74) from three trucking companies operating in Colorado, Kansas, Nebraska, and Texas participated in focus group discussions and completed a short, written survey. In total, 12 focus groups were conducted. In the written survey, 98.7% (*n* = 73) of survey respondents agreed with the statement *“I have pride in the job I do,”* and 97.3% (*n* = 72) agreed with the statement *“Animal well-being is a critical component of my job.”* Additionally, 96% (*n* = 71) of respondents agreed with the statement, *“The training I have received at this job has prepared me well for transporting cattle,”* highlighting the success of current education and training programs. Thematic analysis of focus group transcripts identified ten themes, including (1) Animal Welfare and Safety; (2) Feelings; (3) Human Welfare and Safety; (4) Learning and Training; (5) Money; (6) Opportunities for Improvement Throughout the Supply Chain; (7) Pride, Responsibility, and Care in Job; (8) Specialized Knowledge, Skills, and Commitment; (9) Unique, Positive Characteristics of the Job; and (10) Work Environment. Transporters expressed a strong sense of pride and responsibility in their work and viewed their role as essential to ensuring the well-being of cattle during transportation. However, they also identified several challenges, including ensuring human and animal safety, dealing with inadequate facilities, and a lack of cohesion among different supply chain sectors. As vital links between supply chain sectors, transporters offer unique insights. Listening to their daily observations could lead to improvements in operational efficiency and cattle welfare.

## Teaser

What do cattle transporters feel about their job? They generally enjoy their role working closely with both animals and people. On-the-job training is highly valued by cattle transporters, as is their ability to both stay safe while working and to cultivate a positive working environment.

## INTRODUCTION

The fed cattle supply chain has many sectors working together to produce beef. Generally, cattle are raised on cow-calf operations; they may be backgrounded for some time prior to moving to a feedyard to be finished, and ultimately, they will be shipped to a slaughter facility. Between all these different operations, cattle must be transported; most cattle in the United States will be transported at least once in their lives, and many cattle are transported several times throughout their lifetime (Schwartzkopf-Genswein et al., 2016). Considering transport to slaughter alone, in 2023, over 25 million fed cattle were shipped to federally inspected slaughter facilities in the United States (USDA-NASS, 2024), representing over a half million trailer loads of cattle transported annually. The transporter is critical in ensuring that cattle are delivered safely and humanely to their destination for every load they haul. Transportation is one of the most stressful events that cattle will experience (Chambers and Grandin, 2001), and thus, transporters have a significant responsibility to ensure animal welfare during these important transitions. Depending on the phase of the supply chain (e.g., shipping newly weaned calves, culled cows from auction markets, or fed cattle to slaughter plants), cattle haulers will be faced with different welfare challenges to address.

Animal welfare is important to sustainable beef production as it affects consumer interest, cattle performance, economic potential, and social license to operate (Broom, 2021; Edwards-Callaway et al., 2024). In beef cattle production, interactions between humans and animals are known to impact livestock welfare both positively and negatively (Hemsworth, 2003; Mellor et al., 2020; Mota-Rojas, 2020; Pol et al., 2021; Edwards-Callaway & Sullivan, 2024). The human-animal interactions that occur during transportation are significant, as they almost always occur by unfamiliar handlers, can induce stress or fatigue, and may impact carcass quality (Edwards-Callaway and Calvo-Lorenzo, 2020; Davis et al., 2022; Sullivan et al., 2022). Transporters play a critical role in ensuring high levels of welfare and meat quality, particularly at a phase in the supply chain where handling has significant economic implications (e.g., dollars lost for carcass bruising and carcass discounts for dark cutting). Most literature regarding transportation of fed cattle has focused on environmental factors such as trailer motion, group size, loading density, and transport distance or duration (Swanson and Morrow-Tesch, 2001; Schuetze et al., 2017; Davis et al., 2022). Despite the significant influence transporters have on cattle, few studies have explored transporter perspectives on welfare-related topics; the studies that do exist have focused on topics related to dairy cattle and have been conducted outside the United States (Dahl-Pedersen 2022; Hendricks et al. 2023; Kuo and von Keyserlingk 2023). Within the United States, finished beef cattle transporters’ perspectives about their role in ensuring animal welfare is an important, understudied area of research.

Well-trained personnel enhance the welfare of production animals through practices such as timely euthanasia, low-stress handling, and early recognition of disease, culminating in enhanced stockmanship and management (Grandin, 2018; Edwards-Callaway and Calvo-Lorenzo, 2020). Little is known about the implementation or outcomes of training programs for beef cattle transporters in the United States. The NCBA (National Cattlemen’s Beef Association) Beef Quality Assurance (BQA) program offers a cattle transporter training program for those handling cattle at loading, shipping, and arrival at slaughter facilities. A paucity of literature exists about the impact of training for U.S. cattle transporters, even though driver experience and skill enhance the welfare and meat quality of shipped cattle (Warren et al. 2010; González et al., 2012). The success of training programs for livestock caretakers, which includes transporters, can depend on many factors such as training methods, participant demographics, and previous knowledge base (Coleman and Hemsworth, 2014; Menger et al., 2016b; Daigle and Ridge, 2018; Descovich et al., 2019). Transporter perspectives about their work environments have been documented outside the United States (Dahl-Pedersen, 2022; Hendricks et al., 2023; Kuo and von Keyserlingk, 2023); at times, participants expressed ongoing challenges in compliance or interpretation of regulations, indicating further training needs. It is essential to gather the perspectives of those involved in the care of production animals to design, update, and improve training programs. In addition to cattle welfare and meat quality impacts, job satisfaction, the work community and environment, and occupational safety are essential but widely understudied factors among beef cattle transporters. There is an opportunity to investigate how performing some of the physically or emotionally challenging tasks associated with raising animals for food can influence livestock caretaker job satisfaction and retention, which are critical to workforce stability and industry sustainability.

The objectives of this research were to 1) characterize transporter perspectives and attitudes towards management practices related to cattle care during transportation, 2) characterize transporter perspectives and attitudes regarding training, work environment, and work community, and 3) identify areas for improvement and inform the development of practical strategies (e.g., training and educational resources) to improve animal and transporter well-being.

## MATERIALS AND METHODS

The Institutional Review Board of Colorado State University (CSU) reviewed and approved the study protocols before the project started (Protocol #4419). The study was conducted from August to December 2023.

### Study Population and Recruitment

The population of interest for this study was cattle transporters responsible for transporting finished beef cattle from feedlots to slaughter facilities. Participants represented employees from three commercial trucking companies in Colorado, Kansas, Nebraska, and Texas. Each company had a different hiring structure: Company A employed exclusively company drivers, Company B employed both company drivers and a small proportion of contracted drivers, and Company C contracted all of its drivers. The participating companies were recruited by co-authors of this manuscript, with whom working relationships had been previously established.

### Focus Group Delivery and Format

Research team members facilitated focus group discussions with employees from each company (Company A, n = 29; Company B, n = 20; Company C, n = 25). Focus groups were conducted during annual company meetings or training events. Focus groups were offered in Spanish and English, and participants could choose which focus group they wanted to participate in based on their language preference. Only one focus group was conducted in Spanish (n = 1), and the remaining focus groups were conducted in English (n = 11). The Spanish focus group was hosted by a fully bilingual, native Spanish speaker. The focus group discussions were hosted over lunch to create an informal and comfortable environment and were held in private rooms. Additionally, focus groups with employees were hosted separately from focus groups with supervisor or management-level participants so that everyone could share freely without significant power dynamics. Each focus group ranged from 2 to 12 people, with an average group size of 6 participants. Before any aspect of the focus group began, the facilitator briefly described the objective of the research and obtained verbal informed consent from each person to participate. Participants were also told their participation was voluntary and confidential and that they could leave the room anytime during the discussion.

Before the focus group, participants were asked to answer a brief written survey that took five to ten minutes to complete. The survey contained six author-developed Likert-scale questions about training, job satisfaction, cattle well-being, and work community and environment, in addition to six demographic questions. Surveys were offered in Spanish and English. During the discussion, the facilitator followed a pre-determined list of 22 questions and asked additional follow-up questions only when clarification was needed or to promote discussion amongst the participants. There were three overarching question blocks related to 1) job satisfaction, 2) training, cattle transport, and animal well-being, and 3) work environment and community; the complete list of questions is outlined in **Table 1**. The discussions were audio recorded using the Voice Memos application on an iPad (Apple Inc., Cupertino, CA, USA). In total, 12 focus groups were conducted and ranged between 16 to 50 min; the average focus group length was 29 min in duration (SD = 11.80 min). The focus groups varied in size and thus the total duration was related to the number of people in the group. At the focus group’s completion, all participants were given a $20 cash incentive for their time and participation.

**Table 1.** Focus group questions

Question (Q)	Sub-Question
***Related to job satisfaction***	
Q1: What is your favorite part of your job?	
Q2: What is your least favorite part of your job?	
Q3: What is one thing that would make your job better or easier?	
Q4: What makes you proud of doing your job?	
Q5: Do you see yourself continuing to work in the beef cattle industry?	
***Related to training, cattle transport, and animal well-being***	
Q6: What did you know about transporting cattle before starting your current job?	
Q7: What have you learned about transporting cattle since being at your current job?	
Q8a: What kind of training have you received to do your job?	Q8b: How has your training prepared you for transporting cattle?
	Q8c: Is there more training that would be helpful to you to perform your job?
Q9a: What does cattle well-being mean to you? Define it in your own words.	Q9b: Is that different for other animals, such as pets or horses?
Q10: What is the most critical component of transporting cattle?	
Q11a: What cattle well-being challenges do you experience during transport (e.g., during loading, unloading, etc.)?	Q11b: How do these challenges make you feel?
***Related to work community and environment***	
Q12: What do you like about your current work environment?	
Q13a: How would you describe your work community?	Q13b: Do you spend time together inside or outside of work? Do you want more opportunities to do so?
Q14a: In general, how do you communicate with your coworkers (e.g., call them on the phone, text them, talk to them in person)?	Q14b: More specifically, how do you discuss problems about transporting cattle with your coworkers?
Q15: Do you feel comfortable asking for help when solving a problem related to transporting cattle at work?	
Q16: Is there anything else you would like to share?	

### Statistical Analysis

#### Quantitative analysis.

Respondent answers to the written survey were compiled and entered into a Microsoft Excel spreadsheet (Microsoft Corporation, Washington, DC, USA). Likert-scale and demographic responses were summarized with descriptive statistics.

#### Qualitative analysis.

Recordings of each focus group discussion were transcribed using an online transcription service (Auris AI; Singapore) and then manually reviewed by research team members to ensure accuracy. This process involved a researcher listening to the raw audio file and correcting the AI-generated transcripts for errors, removing any identifying information, and reformatting the transcript so that each speaker (facilitator or participant) was given a new, distinct line of the transcript. Using the same review process, Spanish transcripts were checked for errors and translated by a fully bilingual, native Spanish speaker.

This study employed an inductive framework approach for qualitative analysis (Creswell, 2014). We adopted an interpretive approach that focused on understanding the subjective meanings participants ascribed to their experiences. Our analysis was guided by a constructivist epistemology, recognizing the social construction of knowledge and the impact of the researcher’s perspective, allowing for a deep exploration of participants’ experiences within their broader social context. Eight researchers reviewed all transcripts, familiarized themselves with the data, and independently generated initial codes within the transcripts. As a group, a list of initial themes was created from the patterns found in the initial codes. A detailed codebook with clear theme definitions and examples of each was created and refined iteratively. Following the initial creation of the codebook, three coders conducted intercoder reliability using one transcript. Discrepancies were discussed, and the codebook was reviewed and finalized. After this discussion and revision of the codebook, the coders conducted intercoder reliability on one more transcript achieving greater than 80% intercoder reliability demonstrating strong agreement between coders. Finally, coders were randomly assigned deidentified transcripts and independently coded three or four of the remaining transcripts using the final codebook. Three coders, offering diverse experiences and perspectives, were chosen to code to provide a robust and holistic analysis of the focus group transcripts. The first coder had a Bachelor’s and Master’s degree in Animal Science and was working towards her doctoral degree in the same field, focusing on human and animal well-being in the beef supply chain. The second coder had extensive experience working with caretakers across the supply chain, providing training, conducting audits, and participating in many industry education activities. The third coder earned his Bachelor’s degree in Animal Science and worked as a research associate in the labs of ruminant nutrition and livestock behavior and welfare at the time of this research.

## Results

### Quantitative Results

#### Demographics.

Sample population demographics are described in **Table 2**. Most respondents surveyed were men (95.9%, n = 71), followed by a smaller proportion of women (4.1%, n = 3). A wide distribution of age groups was represented in the study, with survey respondents ranging in age from 18 to 65 and over; the greatest number of respondents reported being within the age ranges of 35 to 44 and 45 to 54 (28.4%, n = 21; 28.4%, n = 21, respectively). A nearly equal number of respondents identified as Hispanic or Latino (48.6%, n = 36) or non-Hispanic or Latino (50.0%, n = 37). Most respondents indicated that English was their preferred language (73.0%, n = 54), followed by 14.9% (n = 11) of respondents preferring to speak two languages. The smallest proportion of respondents indicated that Spanish was their preferred language (10.8%, n = 8). Finally, respondents originated from multiple regions, yet the largest proportion reported being from the United States (70.3%, n = 52) and Mexico (20.3%, n = 15). Respondents were not asked about training status directly but it should be noted that within the industry many companies require Beef Quality Assurance Transportation (BQAT) training of their transporters.

**Table 2.** Survey respondent demographics (N = 74)

Variable	*n*	Frequency (%)
**Language**		
English	54	73.0
Spanish	8	10.8
2 languages^1^	11	14.9
Prefer not to answer	1	1.4
**Ethnicity**		
Hispanic or Latino	36	48.6
Non-Hispanic or Latino	37	50.0
Prefer not to answer	1	1.4
**Gender**		
Man	71	95.9
Woman	3	4.1
**Age**		
18 to 24	6	8.1
25 to 34	15	20.3
35 to 44	21	28.4
45 to 54	21	28.4
55 to 64	9	12.2
65+	1	1.4
Prefer not to answer	1	1.4
**Region of Origin** ^2^		
Africa	1	1.4
Central America	1	1.4
Mexico	15	20.3
South America	1	1.4
United States	52	70.3
No response	4	5.4
**Length of Time Working^3^**		
Less than 6 mo	14	20.3
6 mo to 1 yr	6	8.7
1 to 2 yr	5	7.2
More than 2 yr, but less than 10 yr	28	40.6
10 yr or more	16	23.2

^1^Respondents checked both Spanish and English (n = 10) or English and Portuguese (n = 1) as their preferred languages.

^2^Other regions were offered as choices but were excluded from the table because they were not selected by any respondents.

^3^This question was added after several individuals were enrolled in the study so the total number differs (n = 69).

#### Likert-scale questions.

**Figure 1.**
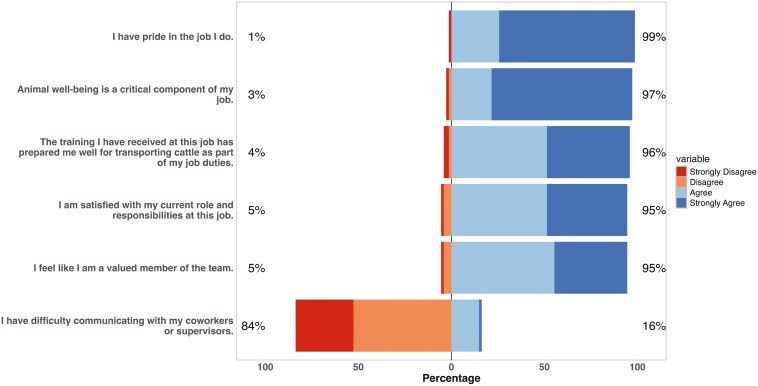
Transporter agreement with Likert scale questions (N = 74).

Responses to agreement questions related to job satisfaction, cattle care, training, and work environment were positive, with most participants expressing strong agreement with most of the statements (**Figure 1**). All but one respondent “Agreed” or “Strongly Agreed” that “I have pride in the job I do” (25.7%, n = 19 and 73.0%, n = 54, respectively). Additionally, most respondents “Agreed” (n = 38, 51.4%) or “Strongly Agreed” (43.2%, n = 32) that they were “satisfied with my current roles and responsibilities at this job.” In response to the statement “Animal well-being is a critical component of my job,” 21.6% of respondents “Agreed” (n = 16), and 75.7% “Strongly Agreed” (n = 56). In response to the statement, “The training I have received at this job has prepared me well for transporting cattle,” the majority of respondents “Agreed” or “Strongly Agreed” with the statement (51.4%, n = 38 and 44.6%, n = 33, respectively). The statement surrounding communication had more variation in responses. In response to the statement “I have difficulty communicating with my coworkers or supervisors,” 52.7% and 31.1% of respondents either “Disagreed” or “Strongly Disagreed” with the statement. In comparison, 14.9% and 1.4% “Agreed” or “Strongly Agreed” (n = 11 and n = 1, respectively).

### Qualitative Results

Thematic analysis of the focus group transcripts identified ten themes, which were: 1) Money; 2) Feelings; 3) Opportunities for Improvement Throughout the Supply Chain; 4) Pride, Responsibility, and Care in Job; 5) Animal Welfare and Safety; 6) Human Welfare and Safety; 7) Specialized Knowledge, Skills, and Commitment; 8) Work Environment; 9) Learning and Training; 10) Unique, Positive Job Characteristics. These themes, as well as their associated definitions and excerpts from the transcripts exemplifying each theme, are shown in **Table 3**. Results from the thematic analysis are presented by each question block (i.e., 1) job satisfaction; 2) training, cattle care, and animal well-being; 3) and work environment and community) are reported below. The quotes included in the text (italicized text in results and discussions) were selected to highlight key ideas and may be edited for clarity and to protect the identity of individuals and companies. The emphasis was on showcasing the richness and connections among themes rather than the number of quotes used.

**Table 3.** Transporter focus group theme definitions, main concepts within each theme, and a sample of quotations from responses to all focus group questions (N = 12). Quotations are chosen to represent themes and may be responses to any number of questions

Theme	Definition & Main Concepts	Example Quotations
**Money**	Related to earning money; making a good living; paychecks; being able to provide/support family; not making enough money/not being paid enough; missing out on loads (resulting in a monetary loss)	*“I like to keep the door closed because I like making money.”* *“I like some of the people, the paychecks.”* *“On these loads, it’s first come, first served. You have to call in for your next load. And if you don’t. You don’t hustle. And you missed out on this load. And now you’re going to [company name] and you missed out on a $300 load.”*
**Feelings**	Related to feelings of the driver, most often in relation to various challenges and setbacks, animal welfare issues, other people, inefficiencies, etc. Examples of feelings may be positive (e.g., happy) or negative (e.g., angry).	*“It’s one of those jobs to where you’re happy.”* *“I think a lot of us, it might frustrate us in the moment. But that doesn’t last. The moment you get in your truck, it’s already done.”* *“Some of us take our job really, really important, and it pisses us off.”*
**Opportunities for Improvement Throughout the Supply Chain**	Related to any component of the supply chain (e.g., the feedlot, dispatch, the plant, the company, supervisors) that impacts their job. Comments could be about anything to do with timing/waiting/inefficiencies, coordination and communication between people and parts of the supply chain, accountability (or lack thereof), and facilities.	*“There’s been times where I’ve sat at the plant and waited for hours. It’s the wait time at the plant that’s really the worst.”* *“There are farms that don’t have much. They only have one chute to load and they send 20 groups an hour. It should be five or six an hour.”* *“Better communication....[next line]...with everyone involved.”* *“Feedlots will push cattle up that they know shouldn’t be going, but they’re trying to sneak them in there. They’re hoping that in the pile of 20 head of cattle that...you didn’t see it. And sometimes we don’t...so they need to be more accountable on that end.”* *“They need to have a little more coordination to receive the cattle in the afternoons.”*
**Pride, Responsibility, and Care in Job**	Sentiments from drivers about their roles and responsibilities during the transportation of cattle; recognizing the importance of their role in ensuring animal welfare; strong sense of responsibility for the cattle in their care; satisfaction in problem solving and executing plans successfully; sense of accomplishment in a job well done; their pride in their job; significance of their contribution to a team and the larger industry.	*“...I haul thousands of head of cattle without injuring them or without hurting them, and I take a lot of pride in that.”* *“But the way I do it is it’s on my watch, so if I’ve got the list and let’s say I’ve got five trucks and I load five trucks, when they get to the other end, everything better get off them trucks because that’s my responsibility.”* *“But all of us actually care about how the animals are treated. It does matter to us the condition of the livestock. And they’re treated well. And we’ve always been taught to give...At least I was. I was always taught to give them the best care you can. Some people say, oh, they’re just going to slaughter. Well, we were still taught to give them the best care you possibly can and treat them the best way you can getting them there.”*
**Animal Well-Being and Safety**	Related to any comments to do with cattle well-being, safety, or anything that may impact cattle well-being and safety specifically. Comments are specific to the animal and cover a broad range of topics (e.g., stress, behavior, handling, quality defects, working with cattle/respecting the cattle, and the physical environment—including the weather, facilities, and driving).	*“...keeping the cattle calm...”* *“I knew we had to get a certain amount of cows to the plant at a certain time, but now I’m seeing that actual handling and how you do it is actually very important.”* *“And if they hit themselves or whatever when they’re hot and stressed, the bruise goes way up on them because they’re hot and stressed.”* *“Treat them with respect.”* *“Different types of cattle very wild and other very calm. One also has to act differently depending on what the cattle are like.”*
**Human Well-Being and Safety**	Related to any comments to do with human well-being, safety, or anything that may impact human well-being and safety specifically. Comments are specific to the human and cover a broad range of topics (e.g., physical environment—including the weather, facilities, and driving; being injured/hurt, dangers of the job, physical toll, etc.).	*“...1550 pounds steer, 105 degree day, working to get them loaded. They’re crazy. He’s crazy. They’re impatient. He’s impatient. And he starts pushing them......and gets hurt......and they’re going to push back. And win. So take a breath. Always. And that’s hard to do.”* *“I didn’t know how dangerous they can be.”* *“And not even just the cattle handling, making it safe on us too. Some of those gates are dangerous too. That’s a big part of it too.”* *“The money is good, but I have an open account at the chiropractor.”*
**Specialized Knowledge, Skills, and Commitment**	These quotes revolve around the unique skills and experiences required for cattle haulers. Refers to the inherent challenges and labor involved in the job. Specialized skills are required to read cattle, load them for comfort, etc. Comments refer to the job not being for everyone/not everyone can do the job, as it requires a certain level of innate ability and experience that cannot necessarily be taught.	*“Cow Savvy”* *“And I was going to say with any livestock, it’s an experience. I don’t think it can be taught.”* *“Not for everybody.”* *“I’ve been doing this for all my life.”*
**Work Environment**	Related to any interactions with people within the company (e.g., other drivers, supervisors, managers), does not include people in other areas of the supply chain; relationships with coworkers, and specific words to describe the environment, such as family, dysfunctional, brotherhood, teamwork, etc.; communication with people at their place of work	*“There is good camaraderie I think in most of us.”* *“Well, it used to. Years ago, it used to be a brotherhood where everybody. When I worked at [company name] everybody helped each other. Now it’s hit and miss. So that’s why I like loading by myself.”* *“We’ve just got like a little family here amongst the drivers. The family. That’s the only people we see during the day.”*
**Learning and Training**	Related to any mention of learning or training. Can be about the method, type, or delivery of training, the relevancy of training, limitations of training, how people learn best (e.g., hands-on, on-the-job), any mention of BQA/BQAT, learning from others, learning something new every day, experience as a teacher.	*“I’ve learned so much since I’ve been here, and the more I learn, the safer our drivers can be, because I can impart that knowledge on them. But just learning about it has been a blast.”* *“And I think it’s whether how much experience you’ve got, you’re going to learn something new every day.”* *“So I’m more of a hands-on or face-to-face, let’s talk about it, learn, show some things. I’m all for that. If there’s more ways to learn out there, I’d be for it.”* *“Sometimes they are totally different worlds for different people. Some people, you do the hands on, they are completely lost. But if you let them read it and they go home and watch it on YouTube, they got it. Where other people, they don’t work that way.”*
**Unique, Positive Characteristics of the Job**	Specific comments regarding distinctive and positive features/attributes/advantages of the job—these comments can be related to travelling new places, variety or work (not boring, new challenges every day), adrenaline rush/excitement, independence—driving/keeping to themselves, enjoying being outside/in the elements, etc.	*“It’s like your own personal office.”* *“I think it’s just about all these cattle, the adrenaline, the rush, you know, just when I started hauling cattle, after I stopped doing it for a while, I couldn’t see myself doing something else, so I went back to hauling cattle. So, I just like the rush, the adrenaline, just the thought of hauling cattle.”* *“And you get to be outside all the time.”* *“Every day is a challenge.”*

### Related to Job Satisfaction

In this section, participants were asked five key questions related to job satisfaction (**Table 1**). These questions explored their favorite and least favorite aspects of their job, what changes could improve or simplify their work, what makes them proud of their role, and their perspectives on their future in the beef industry. In response to these questions, all ten themes emerged with varying frequencies, underscoring the diverse factors contributing to job satisfaction and dissatisfaction among transporters.

#### Positive attributes and job-related successes.

One prominent theme that emerged when discussing the positive aspects of the job was **Work Environment**, particularly the relationships and interactions between colleagues. Participants frequently emphasized their personal and professional relationships as aspects that enrich their work. For example, one participant shared, *“We have each other’s backs. It is like having a really big family,”* while another said, “*My favorite part of the job would be the interaction with the drivers....”* Similarly, the sentiment, *“I think we have a good team”* was echoed by many others. A commonality across these responses was the words used to describe the work environment; drivers frequently mentioned *“family,” “teamwork,”* and *“brotherhood”* to characterize the relationships they have with each other.

Interestingly, the **Work Environment** theme was coded alongside other themes, such as the **Animal Welfare and Safety** theme. These two themes were commonly coded together, highlighting the unique nature of cattle transporters’ work, where interactions with both people and animals play a central role. This unique combination of working with both people and animals in meaningful ways is a key source of job satisfaction for transporters. Statements such as *“working with the people and animals...”* and *“I like the quality of my coworkers – the characters in most of my coworkers, compared to other jobs I’ve had. And of course, I love working with animals, but I think anybody that loves working with animals is gonna be a little better character. And I noticed that I like it”* demonstrate how these themes commonly appeared together in the focus group transcripts.

The **Animal Welfare and Safety** theme related to any comments about cattle well-being, safety, or any factors that impact the animal (e.g., inclement weather, rough handling, poor facilities). Responses coded with this theme were prevalent throughout many of the questions. They included relatively straightforward sentiments such as *“I love working with cattle,” “Hauling cattle,” “I like handling cattle. I always have,”* and finally, *“Playing with the cattle.”* The **Animal Welfare and Safety** theme appeared alongside many other themes, notably **Pride, Responsibility, and Care**. Examples of **Pride, Responsibility, and Care** and **Animal Welfare and Safety** appearing together include *“I take pride in my equipment and like the cattle. I haul thousands of head of cattle without injuring them or without hurting them, and I take a lot of pride in that.”; “You might see us down the road. It’s like, ah, that driver doesn’t care, he’s sitting in a plant. But we actually put the load first.”;* and *“For me, the cows are first and then it’s me.”*

In general, the **Pride, Responsibility, and Care** theme was dominant throughout answers to questions in the job satisfaction block. Responses with this theme were frequently discussed with many other themes, providing more context for why and how transporters feel pride and joy in their work. In many responses to this question, focus group participants reflected on how executing job-related tasks, tackling challenges, and problem-solving issues all lead to a sense of pride for them. Some respondents stated, *“I guess it’s just being able to get home every night. I mean, it’s the rewarding part of having finished what you needed to finish, and we did the best we could and it’s good.”* and put simply—*“Job well done.”;* and *“It is rewarding, too – you got a problem, you put a plan together, you know, you execute it, then you see the end result. It is nice.”* Many respondents discussed being proud of a team and contributing positively to the industry at large. An example of this includes *“COVID helped us more than anything realize what the trucking community does for this nation. So just knowing what the end game is and what we actually do for the whole country.”*

Statements also referred to **Pride, Responsibility, and Care** in the context of the **Specialized Knowledge, Skills, and Commitment** theme. Examples of quotes intertwining these themes include, *“I guess because not just anybody’s willing to do it...when I used to see cattle haulers, I said, man, you know what, that’s a brave dude, or for lack of a better word. Because you know what, you think about them cattle, you can get hurt. But after you do it, ‘hey, I like what I’m doing.’ So, I guess it’s just the fact that not just anybody’s willing to do it,”* and *“I mean, it takes a special person to haul those cattle, right? And to do it right and take pride in getting those cattle to the plant humanely, and that we do treat those animals humanely.”*

Commonly, respondents spoke about **Unique, Positive Job Characteristics**. This theme captured responses where participants highlighted the distinctive and rewarding attributes of being a transporter. Respondents expressed appreciation for being outdoors, enjoying their job’s independence, experiencing something new every day, and having the opportunity to travel to different places. For example, one respondent shared, *“Not a single load is the same, even if you had the same amount of head of cattle. So, it’s just always fun. It’s always exciting. It’s never dull. It’s never boring.”* This idea of experiencing new challenges and never being bored came up in multiple conversations. One respondent shared, *“Every day is a challenge,”* while others echoed, *“It’s a pretty good adrenaline rush”* and *“It’s not boring for sure.”* Other respondents spoke to other positive characteristics, including, *“You get to be outside all the time,” “Places you get to go,”* and *“You get paid to see the country.”*

#### Negative attributes and job-related challenges.

The **Human Welfare and Safety** theme was prevalent in answers pertaining to questions about job-specific challenges. This theme was coded when responses spoke about the physical environment (e.g., weather, facilities, driving) in relation to human well-being and safety, and about the physical toll and inherent dangers of the job. Quotes coded with this theme included statements such as: *“At the same time, the dangers involved and how some drivers don’t take that seriously, and how, in a blink of an eye, our life or somebody else’s life can change. That’s the most frustrating thing.”; “Sometimes loading in the heat.”;* and *“Getting run over and long hours.”* Speaking more to the physical aspect of the job, one respondent shared, *“...by the time I get home and I’m trying to spend time with my kids, if I get on the ground and play with them, I can barely get back up,”* another respondent added, *“The money is good, but I have an open account at the chiropractor.”*

The **Supply Chain Improvement** theme was most common in answers to questions about least favorite job aspects and opportunities to make the job better or easier. However, this theme almost always appeared in conjunction with other themes, particularly about challenges at feedyard facilities and extended waiting times when unloading at the plant. It most commonly overlapped with the **Human Welfare and Safety** and **Animal Welfare and Safety** themes, highlighting the interconnectedness of human and animal well-being, both of which are critical to cattle transporters. In the context of **Human Welfare and Safety**, examples included concerns about facilities, such as: *“And the springs on the tub gate. Some of them are really bad. They’re really dangerous. You let go of that gate and you’re pretty much sitting,”* and *“And not even just the cattle handling, making it safe on us too. Some of those gates are dangerous too. That’s a big part of it too.”* Regarding **Animal Welfare and Safety**, respondents emphasized how poor facility designs negatively affect cattle behavior and proper handling practices. For instance, one participant noted, *“For the cattle to cooperate, we’ve got to have decent facilities..”*

A common thread in the focus group discussions was the issue of truck waiting times at the plant, which frequently appeared alongside mentions and concerns for animal well-being. One transporter explained, “*When you’re moving, it’s fine. But when you’re sitting still and then they hold you for that long, it’s just aggravating for most of us. Not even so much our time or getting them off the truck. Okay, we have to deal with that. That’s part of our job. But all of us actually care about how the animals are treated. It does matter to us the condition of the livestock. And that they’re treated well.”* Another example of this includes, *“...then you get cows on your truck, and you get to a plant, and your expectation is to perfectly not yell, not use your hot shot, all this stuff, but then they have you sit out on the road for four hours. And then we have cows that won’t come off the trailer because they’re hot and exhausted. It has been four hours in 100-degree weather plus a 2-to-4-hour trip.”*

An interesting finding was the frequent mention of the need to address supply chain issues holistically, with many respondents advocating for a more unified effort to ensure animal welfare: “*It’s everybody’s problem because the animal’s welfare is what is supposed to be at the front of the game. It’s a team effort.”* Another respondent shared: *“There could be a bigger emphasis between the transportation side and the feedlot side and the facilities to work together and understand each other. Because right now it’s kind of almost like everybody’s on a different team. It’s almost like we have the stuff that bothers us about them. They have things that bother them about us or whatever. And then in the grand scheme of things, we’re all on the same team. We’re all in the same business and we’re all trying to promote this business in a positive way.”*

Still, respondents commonly referred to the economic losses of supply chain issues, such as potential lost loads and money due to time waiting at the slaughter plant (i.e., the **Money** theme). For instance, one respondent shared, *“I think they should pay us for the entire amount of time. We’ve got to wait thirty minutes before we get paid for our wait time.”* Sometimes, the **Money** theme appeared alone—for example, *“Probably the pay. I’m a salaried employee, and so I’m kind of on call 24-7, and I just don’t feel that pay is compensated for the extra time I put in.”*

While the **Work Environment** theme primarily appeared in responses about what people enjoyed most about their jobs, it also surfaced in discussions of job-related challenges. One respondent captured this complexity well when asked about their least favorite aspect of the job, stating: *“The people. It’s a double-edged sword, it really is.”* When this theme was coded, it most often referred to communication issues among coworkers and with supervisors or upper management. For example, participants made comments such as, *“Communication. Between the drivers and the dispatchers.”* and *“Better communication with everybody involved.”* Another participant shared, *“So, everything we do out there is scrutinized, obviously, and for good reason. But the people typically that are doing the scrutinizing have no idea what we do or what conditions or circumstances we’re stuck with. They don’t know what we have to deal with out there.”* One respondent shared his experience with other drivers and explained some of the challenges related to training and mentoring new drivers: *“When I first started here, I was out at [name of feedlot], and by the time I hit the dock, got all my stuff, and got to the back of the trailer, I was with a bunch of [owner/operators]. They had half of me already loaded, and I had no clue. You know, I didn’t know how many were on the truck, didn’t know what ear tag, you know, nothing. That’s where...if you would slow down and take your time a little more, it’d be a little easier, especially on the newer guys, until they get the hang of stuff.”*

### Related to Training, Cattle Transport, and Animal Well-Being

This block of questions focused on two areas: learning and training (5 questions) and cattle well-being and the challenges related to cattle well-being during transportation (5 questions; **Table 1**). The learning and training questions explored what participants knew about cattle transportation before starting their jobs, what they have learned since, how they acquired that knowledge, whether they found their training helpful, and whether they desired additional training or resources.

#### Training and cattle transport.

The **Animal Welfare and Safety** theme was the most common theme in responses to the training block of questions; this finding highlights how topics surrounding animal welfare and safety are a critical component of learning and training for cattle transporters. In speaking about what they have learned on the job, respondents spoke frequently about animal handling and overall cattle stockmanship (e.g., *“Well, you don’t really have to make...as much of a scene as I thought you did”; “You kind of develop an eye to load them for comfort.”; “Slow down. Faster is not always better.”*). Other respondents discussed what they have learned about cattle needs during transportation specifically; for example, *“I guess more of like the trailer accommodations for them. A little more spacing. We’ve had floors adjusted and changed to ramps that tuck under...so the Holsteins don’t hit their backs.”*

Respondents also spoke about **Human Welfare and Safety**, noting the risks of the job and potential dangers involved with animal handling (e.g., *“I didn’t know how dangerous they can be.”; “We don’t want to break a leg, and we don’t want the cattle to break a leg. It’s both ways.”)*. In reference to questions about what they have learned on the job, respondents often shared insights about the challenges of working in fast-paced environments with multiple moving parts (i.e., **Work Environment** theme). They emphasized the importance of communicating effectively with others and managing both human and animal factors in high-pressure scenarios. An example of this includes, *“I’ve learned from all these feedyards, especially when there’s 10, 15 trucks trying to load at the same time. You’re not only dealing with cows, you’re dealing with drivers, you’re dealing with people. So, you gotta learn how to interact with them because everybody works different.”*

A recurring theme was the culture of ongoing learning and adaptation on the job (i.e., **Learning and Training**). Participants valued the opportunity to observe and learn from more experienced colleagues, noting that this informal learning process was an essential aspect of becoming a better driver. *“My biggest thing is probably watching and learning what other people are doing, and then figuring out your own way of making it easier.”* Along similar lines, another respondent stated*, “I think you learn something every day. I think that’s the beauty of this is we’ve got guys that have way more experience than most of us, but you’ll watch and see them learn something. And that’s what you’re talking about. It’s always different.”*

When speaking about the type of training they received, drivers identified many different types of training and experiences. They spoke frequently about BQA and BQAT training, in addition to practical, hands-on learning experiences. Positive aspects of these trainings included real-life applicability and comprehensive content, while some criticisms pointed to information overload or less relevant content. One respondent stated, *“BQA training. You got to be able to know, to handle the cattle properly, the breeds of cattle. That’s one of the main things that you’ve got to be aware of. As soon as you get into the field, know what kind of cattle you’re going to deal with.”* Another respondent simply said*, “We do BQA.”* Sometimes, participants described the quality of training they received, which often included positive and negative aspects; for example, speaking in reference to BQAT training, this respondent said, *“There’s actually a lot to take away if you can kind of sort through all the information. If you can take what’s applicable and use it and leave what isn’t. Just don’t even let it soak up your brain cells because it don’t apply.”* Speaking in reference to an in-person BQAT training event, this respondent stated, *“This is the best training I’ve had as far as the BQA side goes. And then, the real-life training is, at least with our job, it’s been great. But I’ve also had jobs with real-life training that is awful.”*

Many participants reported receiving training through practical, hands-on experiences. This type of training often involves shadowing experienced colleagues and gradually taking on responsibilities as one’s skills improve. One respondent noted, “*So here, if it’s someone’s first time ever driving a truck, they’re with a driver who sees them for longer. And then if you drove a truck before, they’ll train. And then if they feel comfortable with you being out there, they’ll let them know that he is good to go.”* Respondents emphasized that on-the-job training is a critical component of their learning process, noting that it provides real-world experience that formal training alone may not offer (e.g., *“You get out there and see how [the cattle] really act.”).* One driver spoke about his experiences mentoring new drivers, sharing in part, *“On the way out to every feedlot, I’m going over stuff. ‘This could happen. That could happen.’ Just filling them full of information. But I still, when we get there, I just kind of send them out there and there they go...it’s a very hands-on thing to learn. I can tell them all day long. But until they actually do it, they don’t gain no experience at it. And they don’t understand maybe even what I’m saying sometimes. But, I mean, most of that stuff is just pointers and trying to keep them out of trouble.”* Overall, the emphasis across responses highlights that effective training in this field involves a mix of formal training programs such as BQA and BQAT and practical, on-the-job experience. The focus on **Animal Welfare and Safety** stands out as a key area of concern and training emphasis for drivers.

#### Animal well-being.

In response to questions about animal well-being and the challenges related to ensuring it during transportation, participants frequently emphasized the importance of maintaining cattle health, safety, and overall condition (i.e., **Animal Health and Safety**). Respondents highlighted the need to monitor cattle closely to ensure they are fit for transport, with comments such as *“They’re in good condition,”* and *“Their health to be transported.”* Comments included concerns about bruising and ensuring that all cattle can walk off the trailer as they walked on, reflecting a focus on minimizing stress and injury. For example, *“Safety of the cattle,”* and *“The only thing we see is, are they walking? Are they alive? You obviously have to be conscious of the bruising and all those things. So, I think when you break it down to that level, the biggest thing is, if you load 36 head and they all walk on your trailer, unless there are special circumstances, they better all walk off your trailer.”*

A group of drivers from one focus group engaged in a dialogue about the importance of safety for both humans and animals at every phase of transportation, touching on themes of **Human/Animal Welfare and Safety** as well as **Pride, Responsibility, and Care**. One driver began by sharing, “*Safety for both the people and animals. Because we get people doing silly things back there pushing cattle, and it creates not only a danger for the cows, but a danger for the other people involved.”* Another driver added, *“It’s not just loading or unloading. We’ve got to go down the road. If there’s a curb or a lane shift, we’re top heavy. It’s a lot of responsibility – not just loading or unloading – it’s the middle part of it. For me, the most important stuff is in the middle. I have people pull in front of me...and I can’t stop. Because if I hit the brakes, I might break a cow or something. People don’t understand that. But, loading and unloading is important.”* Facilities were also mentioned when discussing safety; for example, one participant said: “*but there are also some [feedlots] that have very good facilities, but there are others that have very bad facilities where you struggle. So even a person with a little more age struggles. Because if they don’t have good gates, they have to jump over the top. So that’s when it gets dangerous.*”

Along similar lines, respect for cattle and humane treatment were frequently mentioned. Participants spoke about treating cattle with care and avoiding practices that cause undue stress, fear, and discomfort. Some people stated, *“I think you just respect them,”*; *“They’re being treated well,”* and *“Treating them humanely as possible.”* Many respondents highlighted the significance of low-stress handling techniques and cited animal handling as a critical component of animal well-being. Examples of effective animal handling included avoiding excessive electric prod (i.e., hot shot) use and ensuring that cattle are worked smoothly and calmly. Respondents stated, *“The best handling. Getting them from A to B with the less stress and less strain on them,”* and *“low stress environment.”* Another respondent stated, *“But if we’re doing our part to not get them too riled up any more than we have to and get them moving. Just like anything you do, you always have those people. And you have people that think it’s a contest and you’ve got to hit everyone on the way by with your hot shot. It’s not acceptable. And if they’re moving, there’s no reason to bother them. If there going where you want them to, there’s no reason to bother them.”* Respondents often compared cattle well-being to the well-being of other species, such as pets, underscoring the belief that cattle deserve the same level of care and respect; for example, *“Let’s make it their best last ride. You know, I’m an animal lover. I love wildlife, I love all animals, and just because they’re gonna end up on our plate doesn’t mean that they should be treated any differently than your dog at home.”*

Aside from **Animal Welfare and Safety**, the **Pride, Responsibility, and Care**, **Work Environment**, and **Learning and Training** themes were frequently mentioned in respondents’ answers. These themes often provided additional context to respondents’ answers. In one answer to this question, one respondent spoke about animal well-being as a collective responsibility, highlighting the importance of teamwork and camaraderie in ensuring the proper handling of cattle (e.g., *“But if you can tell, it’s the camaraderie. I mean, it really is. Everybody’s got to have everybody’s back. I mean, if somebody’s like, well, I’m going to do this, this, and this, and if you’ve been standing there watching it and you’re like, no, that ain’t going to work. Why don’t you do this instead? Because that second truck tried that, and that ain’t good. I mean, it’s a joint venture. It really is.”).* Another spoke about individual accountability and discussed their personal role in upholding high standards of care, stating, *“It’s my responsibility to make sure that the cattle’s well-being is first and foremost, that they get on safely, and they get off safely.”*

### Related to Work Environment and Community

In this section, participants were asked six key questions about their work community and environment (**Table 1**). These questions explored what respondents enjoyed about their work environment, how they would describe their workplace community, and their communication methods and preferences.

A notable pattern across the focus groups was how transporters described their relationships with coworkers and their community at work. Many respondents characterized their work community with phrases such as, *“Like a brotherhood,” “We’re a family,”* and *“We just got like a little family here amongst the drivers. The family. That’s the only people we see during the day*.” Describing the people he supervises, one manager shared, *“We take care of our employees, because they take care of us. They do most of the work.”* This sentiment was echoed by another driver who shared, *“We have good people. We help each other out.”* This sense of mutual support and camaraderie was frequently highlighted, with descriptions such as, *“There is good camaraderie I think in most of us. We get to the feedlots and sometimes we help each other load.”* Similarly, a respondent shared how they tackle challenges as a team, stating, *“We push each other to do better. You know, when we have challenges, we come up with solutions together.”*

The focus groups repeatedly mentioned communication, again falling under the **Work Environment** theme. Respondents often discussed the importance of resolving conflicts constructively and with a team mentality; for example, *“We all get along, and, you know, if we have a problem, you know, we talk about it like adults. And we find a situation to fix anything that, you know, is wrong.”* Another person continued, *“It’s a family in here. We’re going to get on to each other and yell and whatever, but we always find a way to patch it up, because we all have to work so close.”* When it came to addressing issues at work, one transporter explained the importance of communicating effectively to ensure human safety, drawing upon the **Learning and Training** and **Human Welfare and Safety** themes: *“If you see someone messing up, you don’t try to just scold them. You want to explain to them that, ‘hey, this is how things should be done’, whether or not they want to hear it.”* They continued, *“And so with the newer guys rotating in and stuff like that, that’s something that I try to be anyways. Because some of the guys (not all) will just chew you out and not give you an explanation as to why they’re chewing you out for doing something wrong. I’m one of those guys in particular, you know, I want to take you aside and like, ‘hey man, this is how this should be done. Not like what you were doing – that is going to get you hurt or killed. Like next time, do it like this. It’ll save your life’.”*

The **Learning and Training** theme was inherent to many conversations about the work environment. Often, participants spoke about how their colleagues push them to think differently, learn or refine skills, and adapt to new challenges. One respondent shared more about this, explaining, *“If I’m doing something wrong, I know that I can count on them to tell me. And I try my best not to take it personally, because I feel like, you know, they know better. And the same with me too. I’ll tell [name], ‘hey, we can do this a little different or maybe we’re doing this wrong.’ Just to make things run smoother for everyone.”* In the same conversation, a colleague continues, *“We learn each other’s weaknesses and strengths. I have a hard time getting out of my box and learning something that I don’t know because I have fear of failing. [My colleague] will push me, and he’ll be like,’ you need to figure it out’. But he does it to where I can start getting stronger in that area. So, I mean, we just learn from each other.”*

The concept of community was also frequently discussed with **Human/Animal Welfare and Safety.** Given the inherent dangers of transporting cattle to people and animals, transporters emphasized the importance of looking out for each other and being supportive. For example, one respondent explained, *“When you work with them so often, you know how everybody likes to work...I know how each one of them likes their cows loaded and you just kind of don’t even talk about it. You just learn how to work with each other’s flows.”* Finally, one respondent summed this concept up by saying, *“The majority of the people, they look out for you,”* again highlighting the team mentality among transporters. There were, however, instances when participants spoke about challenges with the work community, again speaking in the context of **Human/Animal Welfare and Safety**; one transporter explained, *“There’s a few [people] I don’t mind loading with. There’s a lot of them I’d rather not, because they’re pushing, they want to go. They want to go fast. You go pushing too fast and constantly hot shot and this and that, you’re gonna get yourself hurt. They’re gonna get hurt. Them cattle are gonna get hurt. So, I prefer to load by myself.”*

Participants spoke more broadly about their work environment and sense of community, touching on a wide range of themes. One prominent theme that emerged from discussions of the work environment and community was **Pride, Responsibility, and Care**. One participant emphasized the importance of pride in their work: *“We try to have fun, to be happy every single day. If you wake up in the morning and aren’t proud to be there—don’t go.”* Another participant reflected on the sense of responsibility inherent in the job, explaining: *“Before load outs and stuff, you laugh, you joke, you giggle. But when it’s time to work, it’s like you flip a switch.”*

## DISCUSSION

Transportation of livestock is a critical component of the meat supply chain. Although many factors can impact cattle during transport (i.e., loading density, transport duration and distance, trailer microclimate, etc., reviewed in Schwartzkopf-Genswein et al., 2012), the individuals hauling the cattle, i.e., the transporters, can have a direct impact on the cattle’s experience. Cattle transporters play a critical yet challenging role in the supply chain, responsible for animal welfare during transport but often lacking the autonomy to make decisions that impact welfare (e.g., fitness for transportation) due to limited ownership or financial responsibility. Despite transporters’ critical role in the supply chain, little research has explored how they perceive their role in the context of cattle welfare, training, job satisfaction, and work environment.

The current study provides information about a group of transporters that haul fed cattle to slaughter as part of their job. In this study, most participants had been working with their current company for over two years with a quarter of participants being in the industry for over ten years. In many of the previous studies on transporters, generally, study participants are experienced, having worked many years in the industry (Burnard et al., 2015; Herskin et al., 2017; Huertas et al., 2018; Valadez-Noriega et al., 2018). Little available data describes the demographics of livestock haulers in the United States. In 2022, there were over two million heavy and tractor-trailer truck drivers in the United States; only 0.3% were involved with animal and aquaculture production (USBLS, 2022). This research provides valuable insight into the perspectives of cattle transporters, a unique group of individuals that play a critical role in the cattle supply chain yet are notably understudied.

Most participants in this study expressed high satisfaction with their roles within the beef supply chain, a positive indicator for the cattle industry. When asked about the most rewarding aspects of their jobs, respondents consistently cited interpersonal relationships, working with animals, and the unique challenges and rewards inherent to their work; participants mentioned the excitement of having different things happen daily, having fun at work, and spending time outdoors. Several studies outside of the United States have investigated factors that motivate farmers to participate in and remain in the farming industry, demonstrating similar intrinsic job factors that contribute to job satisfaction. A study of Norwegian sheep farmers demonstrated that intrinsic motivation, i.e., the doing of an activity for its inherent satisfaction, was highly associated with job satisfaction (Muri et al., 2020). Another study with Swedish dairy farmers similarly demonstrated that intrinsic factors such as having fun at work, feeling pride in your job, and good team spirit were identified as highly important attraction and motivation factors to remain in the dairy profession (Kolstrup, 2012). Transporters in this study also recognized the independence their job affords them; autonomy is often associated with job satisfaction (Hackman and Oldhman, 1976; Humphrey et al., 2007). Although the authors are unfamiliar with existing recruitment strategies for transporters, the positive job characteristics described by participants could inform effective marketing and advertising campaigns for transportation companies. The job’s intrinsic appeal, including the ability to work independently, with animals, and outdoors, may attract individuals to the profession, especially those who are currently not considering a career in transportation.

Previous work demonstrates a positive correlation between job satisfaction and having pride in one’s work (Jin and Guy, 2009). Furthermore, extensive research has explored the factors influencing both job satisfaction and pride in professions that involve emotional labor, i.e., work that requires managing one’s emotions to meet the expectations of a job, such as public service workers, hospitality staff, call center representatives, nurses, and customer complaint specialists (Spinelli & Canavos, 2000; Jin & Guy, 2009; Lewig and Dollard, 2010; Özbezek et al., 2023). Performing this type of work can create a sense of purpose and fulfillment, as it often involves helping others and making a positive impact, leading to greater job satisfaction. Although transportation is not traditionally considered emotional labor, there are some similarities in that transporters are serving multiple different stakeholders (e.g., cattle, feedyard operators, slaughter plant employees, their own employers) and participating in meaningful work, i.e., ensuring cattle are safe and in good welfare during this last step in their lives. Hackman and Oldham’s (1976) job characteristics model provides a valuable framework for understanding the factors contributing to job satisfaction. This model identifies five key elements: autonomy, skill variety, task identity, task significance, and feedback. Transporters in this study emphasized the importance of each of these aspects in their work. For instance, they value their role’s independence (*autonomy*) and the need for a diverse range of skills and knowledge (*skill variety*) to perform their duties effectively. Their job involves a well-defined set of tasks and goals (*task identity*) and has a direct, significant impact on others—particularly cattle and supply chain stakeholders—making their work both meaningful and impactful (*task significance*). Additionally, they rely on their team for support (*feedback*), which is critical for maintaining their performance. These diverse work attributes gave the transporters in this study significant pride in their role, stemming from their critical responsibility for ensuring the welfare and safety of cattle during a crucial stage of meat production.

In the focus groups, transporters were asked what they liked least about their jobs and what would make their jobs easier or better; understandably, there was some overlap in the themes in responses to both questions. Although many different things were shared, a clear trend was related to Supply Chain Improvement. Whether it be facilities, equipment, communication, or scheduling, there was significant mention of improvements in how the supply chain sectors, i.e., the feedyard and the slaughter plant, interact. Transporters are the mechanism that connects the two different sectors together, and thus, it is logical that they would identify challenges on each side that may impact how they perform their jobs. Additionally, part of the frustration expressed regarding supply chain improvements could be related to the fact that transporters have a lot of responsibility but often very little ability to affect change. These individuals aren’t always empowered to make critical decisions for welfare due to contractual agreements or ownership.

Interestingly, when supply chain improvements were mentioned, they were often paired with comments about cattle welfare and human safety. For example, in some focus groups, individuals mentioned that waiting extended periods at the plant to unload cattle can be detrimental to cattle welfare, particularly in hot weather. Despite truck wait time to unload being a component of animal handling audits at slaughter plants (i.e., for an acceptable score, trucks cannot wait for more than 60 min; NAMI, 2021), there are still instances where cattle wait significant periods to be unloaded. A recent benchmarking study of fed cattle arriving at slaughter plants in the United States (Davis et al., 2024b) reported that although, on average, the truck wait time to unload was approximately 30 min, there were extreme wait periods observed, the maximum reported being over nine hours. Increased truck wait time to unload at the plant has been reported to negatively impact cattle mobility (Davis et al., 2024a) and to elevate physiological stress indicators in cattle (i.e., blood cortisol and lactate concentrations, Sullivan et al., 2024).

One other challenge related to the supply chain that was mentioned frequently was the adequacy of feedyard facilities; individuals mentioned this related to their own safety using load outs and handling facilities that were not in working order, but also the cattle’s safety. Working with livestock is a dangerous occupation; the U.S. Bureau of Labor Statistics 2022 annual report indicated that the incidence of injuries and illnesses related to animal production (including livestock, poultry, and aquaculture) was 4.9 recordable events per 100 full-time workers, which was greater than the overall incidence rate for all occupations (USBLS, 2023). An interview study with Danish cattle handlers who had been injured while working with cattle reported that most injuries sustained by study participants could have been prevented with facility changes (Nielsen and Norup, 2024). Additionally, poor facilities have been identified as a factor negatively impacting cattle injury and/or bruising (Weeks et al., 2002; Mendonça et al., 2016). In a survey study focused on the ease of sheep handling with transporters in Australia, study participants unanimously reported that facilities were the most significant factor impacting sheep handling (Burnard et al., 2015). Improving and continually managing cattle handling facilities could significantly impact transporter and cattle safety.

Regarding the discussion about transporter safety, human safety was frequently mentioned in response to what participants had learned about working with cattle since they started their jobs. Many focus groups recounted that before beginning to haul cattle, there can be a lack of understanding about how working with cattle can be dangerous. Human safety should be a critical component of training programs for working with livestock. Nielsen and Norup (2024) concluded from their interview study with cattle handlers who had been injured while on the job, that stockmanship training was crucial to improving safety; interestingly, the majority of participants in that study indicated that they thought their reported injuries could have been prevented if they had changed their behavior (e.g., moved less rapidly, did not take a risk, or moved more calmly). Ramos et al. (2018) reported that less than half of the feedlot workers in their study had received health or safety training at their place of work, and the vast majority were interested in learning about their jobs’ health and safety hazards. Similar literature does not exist for cattle transporters. The availability of and implementation of livestock stockmanship training for transporters varies across areas of the world (e.g., Spain—Miranda-de la Lama et al., 2010; Australia—Burnard et al., 2015; Denmark—Herskin et al., 2017; Ghana—Mogre et al., 2024). Many cattle transporters in the United States have been BQAT certified; 2024 statistics from NCBA indicate that over 20,000 individuals have become BQAT certified online, and over 2,500 trainings have been completed in person (personal communication, J. Fitz-Simmons). In addition to recognizing the value of the BQAT program, the current study participants also indicated that animal welfare and topics such as low-stress and humane handling were other areas they had learned more about since they started hauling cattle. The cattle industry has promoted the benefits of using low-stress handling techniques for decades (Grandin, 1997; Stookey and Watts, 2014), and many of the producer-facing industry education programs (e.g., BQA, 2019) provide both written materials, video, and in-person opportunities to learn from industry experts about the how and why of humane cattle handling. It is evident from this study that transporters learn from these opportunities, and the beef industry should continue to provide them.

A critical aspect of learning which was shared in the focus groups was the importance of hands-on and experiential opportunities for training and continuing education. Many participants talked about learning from more experienced transporters when they started their jobs as a crucial part of their training. Other studies have demonstrated that livestock transporters learn from working with others, such as family members or other transporters within their companies. An interview study with Mexican cattle transporters reported that over 30% of the transporters learned how to be a cattle hauler from a colleague at work, and nearly half reported learning from a family member (Valadez-Noriega et al., 2018). Similarly, Burnard et al. (2015) reported that the majority of sheep transporters in their study gained livestock handling experience on a family farm and the others learned on the job. Herskin et al. (2017) also reported that drivers in their study shared that peer-to-peer learning was an important mechanism for them to learn about fitness for transport. Research consistently indicates that livestock caretakers are seeking expanded training opportunities in various areas with a desire for practical, in-person learning (Burnard et al., 2015; McGee et al., 2016; Menger et al., 2016a; Simpson et al., 2020; Denis-Robichaud et al., 2023). The current industry training programs, primarily the NCBA BQAT program, were referenced many times across focus groups, with participants highlighting the benefits which the program and associated events have brought them. The simultaneous recognition of the value of current training programs and the need for additional opportunities presents an ideal context for developing and implementing more programs facilitated by local, state, and national cattle industry representatives.

Ensuring animal welfare is an integral part of a transporter’s job, and their acknowledgment of and recognition of this role and responsibility was apparent throughout the study; the vast majority of participants agreed that animal welfare was a critical component of their job. This is a positive finding as transporters are a critical link between supply chain sectors in the beef industry. Additionally, the theme of animal welfare was found across numerous focus group questions. Study participants were asked directly what animal well-being meant to them, and responses varied widely, including mentions of animal health, condition for transport, injury, and meat quality defects (e.g., bruising). They recognized their role in reducing stress for the cattle during this critical period. Other studies with transporters have demonstrated similar care and empathy toward animals (Burnard et al., 2015; Valadez-Noriega et al., 2018). In a survey study of sheep transporters in Mexico, slightly over half of the transporters agreed that transportation impacted animal welfare. This study identified similar transport-related welfare issues as the current study, such as bruises and injuries (Pulido et al., 2019). It is well documented that human attitudes towards livestock can impact how they interact with animals and thus animal welfare (Hemsworth et al., 1989; Waiblinger et al., 2002; Coleman et al., 2003; Kauppinen et al., 2012; Leon et al., 2020; Pol et al., 2021). While this study provides evidence of transporters’ awareness of their impact on cattle welfare, future research could benefit from a more in-depth examination of their specific attitudes towards livestock and approaches to animal handling.

The work environment was a job aspect that the study participants liked the most, and they often mentioned explicitly the people they worked with. Across the focus groups, it was clear that they appreciated a team culture and a sense of camaraderie among the drivers; even though the transporters in this study were often working independently, they still expressed that they had a strong network of support from other drivers, which was a highly positive component of their work environment. In addition to job autonomy, as mentioned previously, social support (i.e., getting assistance from co-workers and/or finding friendships at work) is another critical factor influencing job satisfaction (Humphrey et al., 2007), and this support was noted as a positive component of study participants’ work environments. Communication was also mentioned when discussing the work environment; the study participants mentioned the importance of resolving conflicts constructively and tackling some of those challenges as a team. Although not articulated as such, the study participants touched on the organizational culture of their work environments. Organizational culture has been defined by many (Pettigrew, 1979; Schein, 1990; Claver et al., 2001; Ravasi and Schultz, 2006), but Deal (1991) has written about it as “the human invention that creates solidarity and meaning and inspires commitment and productivity,” a definition that accurately captures the feelings shared by transporters in the current study. There is a large body of research demonstrating the association between organizational culture, job satisfaction, and other related factors (Lund, 2003; MacIntosh and Doherty, 2010; Belias and Koustelios, 2014; Akpa et al., 2021), but the work has not been specifically focused on the transportation industry. Although not specific to transporters, there has been some relevant work exploring safety, stress, and other critical components of working conditions on feedyards with a specific focus on Latino/a immigrant workers (Ramos et al., 2018, 2019, 2021). Future research could delve deeper into the specific aspects of organizational culture that contribute to job satisfaction among livestock transporters, providing valuable insights to enhance employee well-being and performance.

At the completion of the focus groups many respondents expressed appreciation for the opportunity to share their perspectives. It is clear from this study that transporters have immense pride in their work and feel a strong sense of responsibility for their roles in the beef supply chain, particularly in ensuring high standards of cattle care. As vital links between supply chain sectors, transporters offer unique insights. Listening to their daily observations could lead to improvements in operational efficiency and cattle welfare. This study is one of the first to explore the perspectives of cattle transporters in the United States, aiming to enhance both their job satisfaction and the well-being of the animals in their care. Additionally, the positive aspects of the job highlighted in this study, such as the ability to make a meaningful impact on cattle welfare, could be a valuable tool for attracting new individuals to the transportation industry. Future research should build on these findings by creating more continuing education and training opportunities. Developing and administering a validated job satisfaction scale specific to transporters would provide an opportunity for quantitative analysis and comparisons across different demographics. Additionally, there is a lot of opportunity for direct assessment of transporter training programs (e.g., BQAT) on transporter knowledge, attitudes, and handling practices. Future research could evaluate mechanisms targeted at improving communication and coordination between feedyards, transporters, and slaughter plants in addition to some of the other challenges addressed in this research.
